# Clinical analysis of severe *Chlamydia psittaci* pneumonia: Case series study

**DOI:** 10.1515/biol-2022-0698

**Published:** 2023-09-26

**Authors:** Xi Zheng, Chonghao Wu, Bing Jiang, Guangmei Qin, Ming Zeng

**Affiliations:** Department of Respiratory and Critical Care Medicine, Yongchuan Hospital Affiliated to Chongqing Medical University, 439 Xuanhua Road, Yongchuan District, Chongqing, 402160, China

**Keywords:** *Chlamydia psittaci*, severe pneumonia, mNGS

## Abstract

The clinical characteristics and diagnosis of ten cases with severe *Chlamydia psittaci* pneumonia were analyzed. Ten patients had high fever, cough, or diarrhea, and all had a history of contact with birds or poultry. The white blood cell count of the patients was normal or slightly increased. The percentage of neutrophils (*N*%) and C reactive protein of the patients were significantly increased. Chest computer tomography showed patchy consolidation of both lungs, with one-sided lung lobes prominent, and bronchial inflation signs. All the patients were admitted to the intensive care unit due to respiratory failure. Nine patients needed ventilator-assisted ventilation therapy, and one patient needed high-flow oxygen therapy. All patients had sepsis, and five patients developed septic shock. The patients were diagnosed with severe *C. psittaci* pneumonia by clinical manifestations and contact history. After timely adjustment of tetracycline-based treatment, eight patients recovered and were discharged, and two patients died of septic shock and respiratory failure. Patients with poultry contact should be cautious toward *C. psittaci* pneumonia. A better method for the detection of *C. psittaci* is metagenomic next-generation sequencing. Its examination can shorten the diagnosis time. In a later stage, large-sample research is needed to guide clinical diagnosis and treatment.

## 1 Introduction

Community-acquired pneumonia (CAP) is the most common infectious disease of the respiratory system. The different clinical characteristics of CAP in patients determine the severity of pneumonia, which can provide the choice of treatment, help to shorten the course of treatment, reduce the risk of death, and reduce the economic burden of patients [1]. *Chlamydia psittaci* can infect humans and a variety of animals and can be caused by direct or indirect human exposure to aerosols, feathers, dust, or respiratory secretions from the feces of infected birds [2]. In recent years, it has been reported in the literature that *C. psittaci* pneumonia accounts for approximately 1% of CAP [3]. The clinical manifestations of *C. psittacosis* pneumonia are diverse, and the typical clinical manifestations are high fever, chills, headache, myalgia, cough, and pulmonary infiltrative lesions [4]. The general symptoms are similar to those of a cold or respiratory infection. Although pneumonia is the most common manifestation, severe patients often accumulate pathogens in the liver or heart, leading to multiple organ dysfunction [5]. Conventional inspection methods cannot identify pathogens, but metagenomic next-generation sequencing (mNGS) technology can detect rare pathogens early, thereby reducing unnecessary antibiotic use and shortening hospitalization time [6]. This article analyzes and summarizes the clinical characteristics of severe *C. psittacosis* pneumonia, hoping to enable clinicians to have a better understanding of *C. psittacosis* pneumonia, which has certain guiding value for clinical work.

## 2 Methods

### 2.1 Study design

A retrospective analysis of ten patients with severe *C. psittaci* pneumonia admitted to Yongchuan Hospital Affiliated with Chongqing Medical University from May 2019 to May 2021. Demographic and clinical data of patients were collected, including age, gender, epidemiological contact history, clinical manifestations, laboratory data and imaging data; clinical prognosis and follow-up data were collected. The study was approved by the Ethics Committee of Yongchuan Hospital Affiliated to Chongqing Medical University, and all data were processed anonymously.


**Informed consent:** Informed consent has been obtained from all individuals included in this study.
**Ethical approval:** The research related to human use complied with all the relevant national regulations, and institutional policies, and is in accordance with the tenets of the Helsinki Declaration, and has been approved by the Ethics Committee of Yongchuan Hospital Affiliated with Chongqing Medical University.

### 2.2 Observation record indicators

The observation record indicators are as follows: (1) epidemiological data; (2) Clinical data: age, gender, underlying diseases; clinical symptoms: fever, cough, diarrhea, time from onset to admission, time to respiratory failure, intensive care unit (ICU) hospitalization time, total length of hospital stay, and assisted ventilation, and prognosis; (3) laboratory data: oxygenation index (OI); infection indicators: white blood cell (WBC), C reactive protein (CRP), procalcitonin (PCT), platelets; blood biochemical indicators: alanine aminotransferase (ALT), aspartate transaminase (AST), lactate dehydrogenase (LDH), creatine kinase (CK), sputum culture, bronchoalveolar lavage fluid culture, and mNGS etiological results; and (4) electron computed tomography.

### 2.3 Diagnosis and definition

According to the 2016 edition of Chinese guidelines for the diagnosis and treatment of adult CAP [1], CAP is divided into non-severe CAP and severe CAP. Severe CAP criteria included the following: those who meet one of the following major criteria or ≥3 minor criteria; the major criteria are as follows: (1) mechanical ventilation with tracheal intubation is required and (2) septic shock still needs vasoactive drug therapy after active fluid resuscitation. Secondary criteria included the following: (1) respiratory rate ≥30 breaths/min; (2) OI ≤250 mmHg (1 mmHg = 0.133 kPa); (3) multilobar infiltration; (4) disturbance of consciousness and/or disorientation; (5) blood urea nitrogen ≥7.14 mmol/L; and (6) systolic blood pressure <90 mmHg requires active fluid resuscitation. Sepsis is defined by the 2016 Sepsis 3.0 guidelines as a dysregulated body response to infection, resulting in life-threatening organ dysfunction [7]. Septic shock is defined as sepsis as circulatory and cellular metabolic disturbances that are severe enough to increase mortality and require vasopressors to maintain mean arterial pressure of 65 mmHg (1 mmHg = 0.133 kPa), when hypovolemia is excluded, and blood lactate >2 mmol/L in the absence of hypovolemia.

## 3 Results

### 3.1 Patient characteristics

Six men and four women with severe *C. psittaci* pneumonia were identified in the age range of 43–72 years (Table 1). All patients were positive for *C. psittaci* DNA fragments by mNGS from bronchoalveolar lavage specimens. Three patients had a history of contact with birds, and seven patients had contact with poultry. Three patients had a history of diabetes mellitus, one patient had a history of coronary heart disease, two patients had a history of hypertension, and one patient had a history of chronic obstructive pulmonary disease. Eight patients started with high fever, accompanied by chills, remittent fever higher than 39°C, cough, no obvious expectoration, accompanied by fatigue, limb muscle pain, two patients with diarrhea accompanied by heart, vomiting, followed by fever, and all patients developed progressive breathing difficulty (Table 1).

**Table 1:** Demographic and clinical characteristics of the ten patients

Case	Age (years) and gender	Onset time (day)	Underlying diseases	History of avian or poultry contact	Symptoms
1	67 F	5	Diabetes	Long-term poultry raising	Fever, dyspnea, and diarrhea
2	64 M	4	Diabetes	Frequent visits to the market	Fever, cough, and vertigo
3	48 M	10	Hypertension	Long-term poultry raising	Fever, cough, and expectoration
4	66 F	4	COPD	Long-term poultry raising	Fever, cough, and weakness
5	66 F	5	None	Swallow’s nest at home	Fever, cough, and diarrhea
6	43 M	20	Diabetes	Pigeon breeder	Fever, cough, and diarrhea
7	72 F	2	Coronary heart disease	Contact with plague chickens	Fever, cough, and expectoration,
8	50 F	2	None	Poultry butcher	Fever, dyspnea, and vomiting
9	54 M	5	None	Long-term poultry raising	Fever, cough, and diarrhea
10	69 F	7	Hypertension	Kept parrots at home	Fever and diarrhea

On admission, the WBC was generally normal or slightly elevated (7.6 ± 1.91) × 10^9^, the percentage of neutrophils *N*% was 87.67 ± 11.6%, the procalcitonin (PCT) level increased to 3.0 ± 4.2 ng/mL, and the level of CRP increased to 176.2 ± 66.5 mg/L. One patient had decreased platelets; eight patients had increased AST and ALT; and five patients had increased CK and LDH (Table 2). All ten patients had respiratory failure, and the OI was less than 200 mmHg, of which three patients had OI <100 mmHg. All patients were evaluated as having severe pneumonia of *C. psittaci*, with an average acute physiology and chronic health evaluation II score of 22 (21–23), and a Sequential Organ Failure Assessment Score (SOFA) was 8 (6–9) points; all patients suffered from sepsis, of which five patients had septic shock. The time from onset to respiratory failure was 5 (4–7.5) days, eight patients received invasive ventilator-assisted ventilation after tracheal intubation, one patient received non-invasive ventilator-assisted ventilation, and one patient was given high-flow oxygen therapy, breathing machine support time was 10 (9–15) days. The length of stay in the ICU was 8.5 (4.5–15.8) days, and the total length of stay was 18 (11.75–25) days (Table 3).

**Table 2:** Laboratory parameters of the 14 patients

Case	WBC (×10^9^/L, 3.5–9.5)	CRP (mg/L, ＜10)	PCT (µg/mL, 0–0.5)	*N* (%, 40–75)	PLT (×10^9^/L, 100–300)	CK (U/L, 55–170)	LDH (U/L, 313–618)	ALT (U/L, 9–50)	AST (U/L, 15–40)
1	9.3	213	1.56	97.3	104	5,317	2,182	302	566
2	5.4	147	3.99	91.4	78	256	1,219	70	149
3	6.2	16.6	0.07	71.8	486	81	925	176	216
4	6.2	124.4	0.83	91.8	121	69	601	94	56
5	8.2	188.1	5.56	97.6	381	71	1,338	120	192
6	11	234	0.98	89.3	198	20	2,299	322	538
7	6.3	208	1.87	61.9	237	43	381	27	30
8	10	254	1.38	88.8	147	194	232	41	42
9	7.1	188.1	13.79	92.1	138	528	3,356	149	468
10	6.3	193	1.77	94.7	195	225	2,252	513	828

WBC: white blood cell count; CRP: C-reactive protein; PCT: procalcitonin; *N*%: neutrophil percentage; CK: creatine kinase; LDH: lactate dehydrogenase; ALT: alanine aminotransferase; and AST: aspartate aminotransferase.

**Table 3:** Treatment and outcome of ten patients with severe *C. psittaci* pneumonia

Case	OI (400–500)	SOFA	APACHE II	Sepsis	Septic shock	Supportive treatment	Supportive time (days)	ICU admission time (days)	Length of hospital stay (days)
1	70	9	24	Yes	None	Mechanical ventilation	7	15	25
2	187	8	23	Yes	None	Mechanical ventilation	7	11	23
3	150	5	18	Yes	None	High flow oxygen	—	3	14
4	150	5	22	Yes	Yes	Non-invasive ventilator	5	5	16
5	102	7	21	Yes	Yes	Mechanical ventilation	19	20	20
6	132	7	21	Yes	Yes	Mechanical ventilation	10	15	26
7	164	8	23	Yes	None	Mechanical ventilation	10	18	25
8	200	6	16	Yes	None	High flow oxygen	—	6	16
9	84	8	26	Yes	Yes	Mechanical ventilation	5	5	5
10	63	10	21	Yes	Yes	Mechanical ventilation	2	2	2
Median (range) or (mean ± SD)	130.2 ± 48	8 (6–9)	22 (21–23)	—	—	—	10 (9–15)	8.5 (4.5–15.75)	18 (11.75–25)

OI: oxygenation index; SOFA: Sequential Organ Failure Score; APACHE II: Acute Physiology and Chronic Health Score II.

### 3.2 Bronchoscopy and imaging features

**Figure 1:**
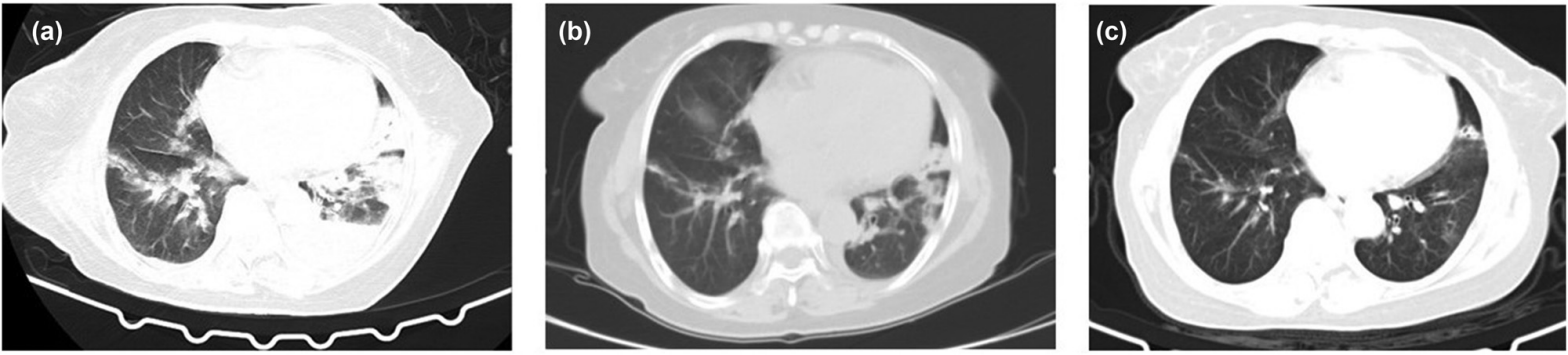
The CT image of case 1. Picture (a) (2020.10.19) is admission D12 and DOX D4 is used, showing multiple patchy and nodular increased density shadows in both lungs, the left lung is obvious, and the left pleural effusion. Picture (b) (2020.10.28) shows admission D22, DOX D14, and the lesions in both lungs were obviously absorbed. Picture (c) (2020.12.07) was reviewed 1 month after discharge, and the lesions in both lungs were completely absorbed.

**Figure 2:**
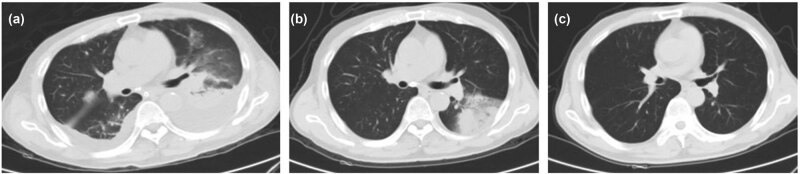
The CT image of case 2. Picture (a) (2020.12.18) is the admission D1, pleural effusion in both lungs, exudation, consolidation, and exudation in the lower lobe of the left lung. (b) Picture (2020.12.29) is the admission D12, Dorsey cyclin D4, the pleural effusion in both lungs was completely absorbed, and the lesions in the lower lobe of the left lung were significantly reduced. Picture (c) (2021.03.11) was reviewed 2 months after discharge, and no obvious lesions were found in both lungs.

**Figure 3:**
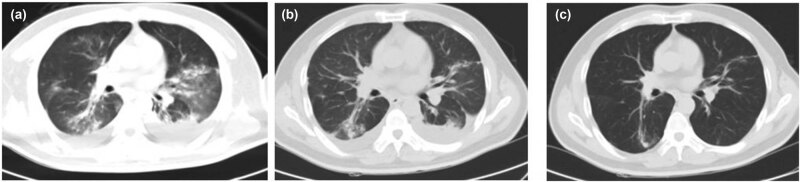
CT image of case 6. Picture (a) (2019.07.23) is admission D10, DOX D9, multiple infectious lesions in both lungs and bilateral pleural effusion. Picture (b) (2019.08.06) is admission D27, DOX sulfur D26, and the infection lesions in both lungs were reduced. Picture (c) (2019.08.27) was re-examined 3 weeks after discharge, and the lesions in both lungs were significantly absorbed compared to before.

**Figure 4:**
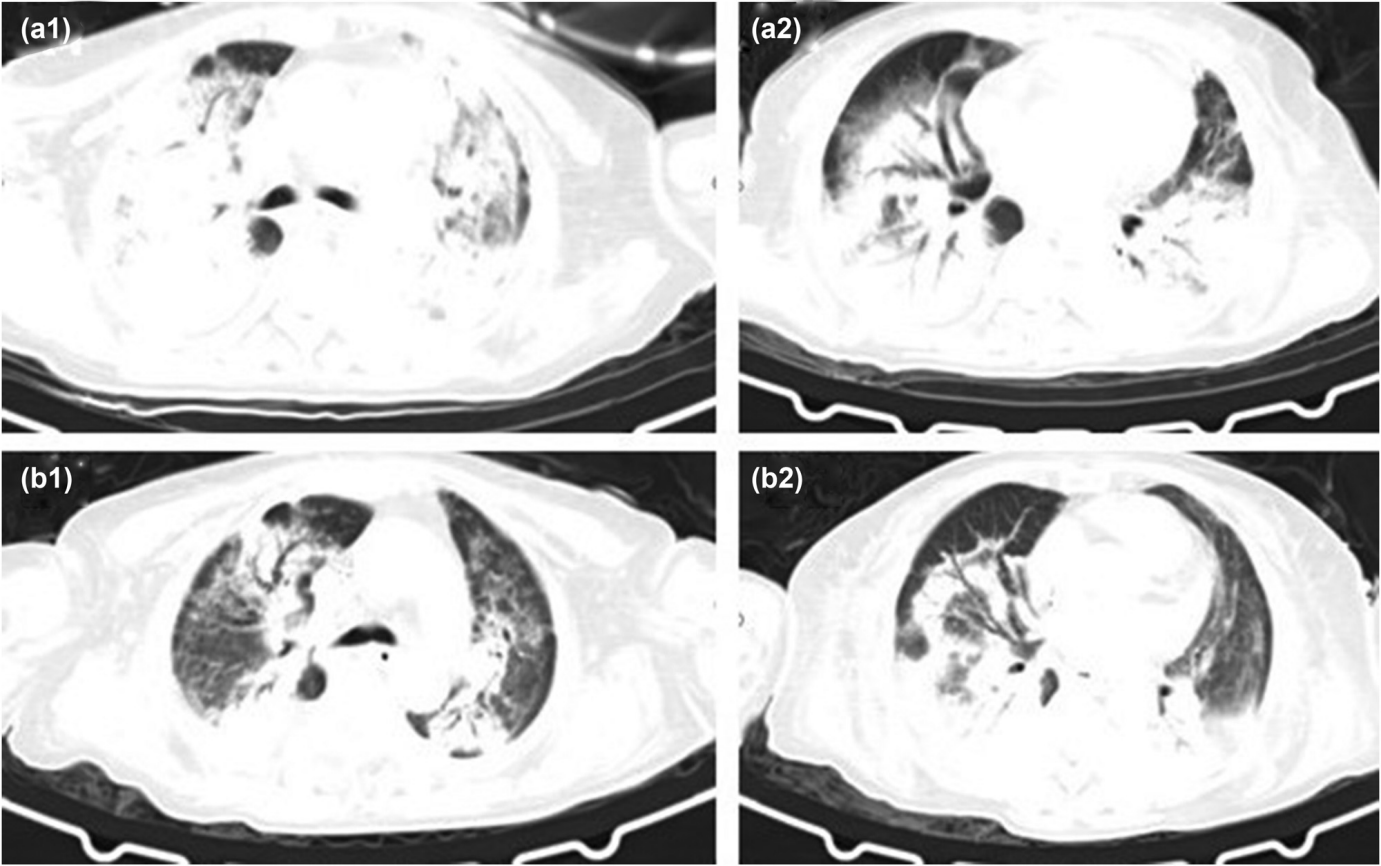
CT images (a1) and (a2) of case 5 (2020.12.26) are on the first day of admission, with multiple patches and patchy slightly high-density shadows and consolidation shadows in both lungs, with air bronchus sign, and bilateral pleural effusion. (b1) and (b2) (2021.01.11) show the admission D16, DOX D13, and the bilateral lung infection lesions are less than before.

**Figure 5:**
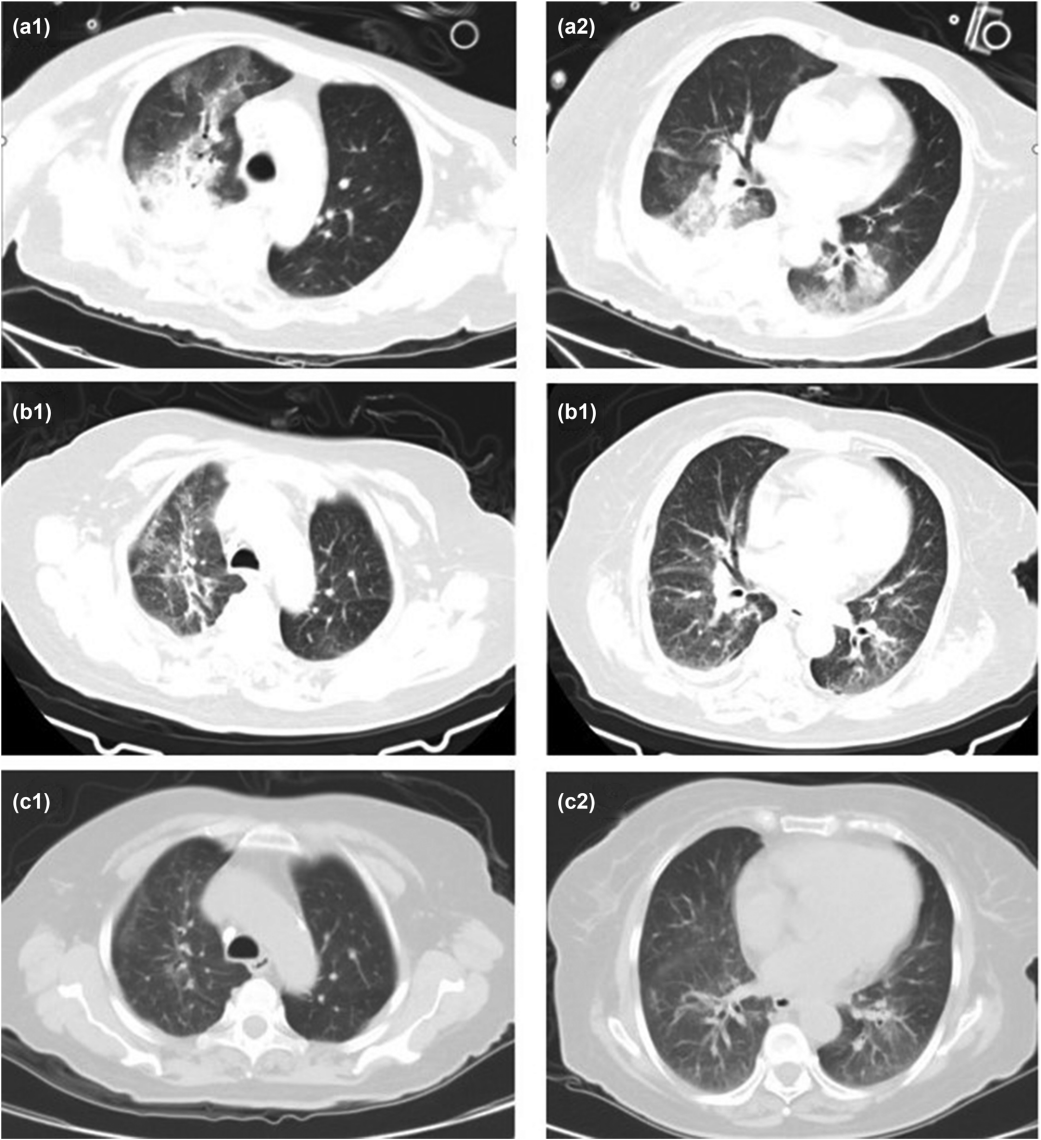
CT images (a1) and (a2) of case 7 (2021. 02.23) are admission D8, DOX D1, bilateral lung infection. (b1), (b2) (2021.03.04) are admission D18, DOX D11, and the bilateral lung infection lesions were slightly reduced. Pictures (c1) and (c2) (2021.03.27) were re-examined 2 weeks after discharge, and the bilateral lung lesions were significantly absorbed.

Bronchoscopy showed that the bronchus was mainly manifested as airway mucosal congestion with edema, and a small amount of white or yellow sticky sputum was seen in the segmental bronchi. The chest computer tomography (CT) of eight patients showed multiple patches and flaky slightly high-density blurred shadows and consolidation shadows in both lungs, and the edges were not clear. The unilateral lung was marked with high-density exudation and consolidation shadows, and some lesions were also seen in the bronchus. Inflatable sign: one patient presented with unilateral large patchy consolidation in the upper lobe of the right lung. Only one patient with chronic obstructive pulmonary disease showed multiple patchy ground-glass opacities and grid opacities in the right lung. Six patients had bilateral pleural effusion, three patients had unilateral pleural effusion, and one patient had no pleural effusion. After effective treatment in eight patients, the inflammatory lesions were basically completely absorbed, and there was no residual organized fibrous tissue. Two patients died within a short period of time in hospital due to critical condition, and CT scan was not repeated (Table 4 and Figures 1–5).

**Table 4:** Bronchoscopy and imaging features of ten patients with severe *C. psittaci* pneumonia

Case	Bronchoscope	CT signs	Lobar lesions	Pleural effusion	Outcome
1	Left lung mucosa is congested with leukoplakia and mucous sputum	Multiple patchy lungs in both lungs and the left lung was obvious	Double	Left	Absorb
2	Airway mucosa is congested with thick yellow sputum and bloody secretions	Multiple patchy shadows were seen in both lungs, with consolidation in the lower lobe of the left lung	Double	Double	Absorb
3	Right bronchial mucosa hyperemia with thin white secretions	Multiple patchy ground-glass opacities and grid opacities in the right lung	Right	None	Absorb
4	Tracheal mucosa swelling and congestion, sticky phlegm	Patchy high-density shadows were seen in the medial segment of the right middle lobe and the upper and lower lobes of the left lung	Double	Left	Absorb
5	Bilateral tracheal mucosa hyperemia, white jelly-like sputum can be seen	Multiple plaques and consolidation shadows in both lungs	Double	Double	Absorb
6	Yellow sticky phlegm	Scattered patchy and flaky high-density shadows were seen in both lungs	Double	Double	Absorb
7	Airway congestion with visible bloody discharge	Multiple patches in both lungs	Double	Double	Absorb
8	Viscous secretion in the right upper lobe bronchus	Patchy consolidation with air bronchus sign in the right lung	Right	Double	Absorb
9	Viscous yellow sputum can be seen in each segment of the lower lobes of both lungs	Multiple patchy shadows in both lungs	Double	Left	Unhealed
10	Mucosal congestion, white foamy sputum in both lungs	Multiple patches and lamellar consolidations in both lungs	Double	Double	Unhealed

### 3.3 Etiological results

All patients underwent bronchoscopy, and bronchoalveolar lavage fluid samples were collected and sent for mNGS examination. The time from admission to the detection of *C. psittacosis* was 6.5 days. All patients underwent sputum culture after admission, and the first sputum culture results were negative, and no bacterial growth was found. *Acinetobacter baumannii* was cultured in the sputum of five patients (Table 5).

**Table 5:** Treatment and outcome of 14 patients with severe *C. psittaci* pneumonia

Case	Time from admission to diagnosis (days)	mNGS *C. psittaci* reads (*n*)	mNGS other pathogens read (*n*)	Culture results	Antibiotics before diagnosis	Antibiotics after diagnosis	Length of hospital stay (days)	Outcome
1	6	7,354	*Corynebacterium gluconolytica* (286)	*Acinetobacter baumannii*	IPM + LVX	IPM + DOX	25	Survived
2	6	895	None	*Acinetobacter baumannii*	IPM + MXF	IPM + DOX	23	Survived
3	9	1,893	None	None	MEM	CSL + DOX	14	Survived
4	6	2,634	*Staphylococcus aureus* (185)	None	TZP + TGC	TZP + TGC	16	Survived
5	5	952	None	*Acinetobacter baumannii*	TZP + MXF	TZP + DOX	20	Survived
6	4	2,972	None	*Acinetobacter baumannii*	IPM + MXF	LVX + DOX	26	Survived
7	7	4,670	*Candida albicans* (703)	*Acinetobacter baumannii*	TZP + TGC	IPM + DOX	25	Survived
8	4	490	*Aspergillus fumigatus* (2)	None	TZP + MXF	TZP + DOX	16	Survived
9	5	837	*Streptococcus gallus* (149)	*Acinetobacter baumannii*	IPM + LVX	IPM + DOX	5	Death
10	3	3,835	*Propionibacterium acnes* (4)	None	TZP + MXF	NA*	2	Death

DOX = doxycycline, IPM = imipenem, LVX = levofloxacin, MEM = meropenem, MXF = moxifloxacin, NA = not applicable, TZP = piperacillin-tazobactam, TGC = tigecycline, CSL = culbactam and cefoperazone. *The patient died before the mNGC results were reported.

### 3.4 Treatment

After admission, all patients were given empiric anti-infective therapy with carbapenems and quinolones according to the adult CAP guidelines [8]. The bronchoalveolar lavage fluid was collected and sent to mNGS for examination. It took 48–72 h from mNGS collection to reporting results. When *C. psittacosis* pneumonia is diagnosed, tetracyclines with higher intracellular activity are selected [9], so that the anti-infection regimen is adjusted to doxycycline (DOX) or tigecycline (TGC). The patient was also treated with imipenem, cilastatin, piperacillin sodium, tazobactam, cefoperazone sodium, sulbactam sodium, or levofloxacin for anti-infection (Table 5).

### 3.5 Prognosis

After the addition of tetracycline (DOX or TGC) in seven patients, their body temperature decreased, their symptoms improved, and they finally recovered and were discharged from the hospital. One patient still had recurrent fever after adding DOX, and the chest CT showed new lesions. After resubmission of mNGS, the patient was complicated with *Candida albicans* infection. Therefore, after adding voriconazole, the patient’s body temperature gradually decreased. One patient died because of bacteremia, septic shock, and DOX treatment, and another patient died due to uncorrectable respiratory failure and complications such as septic shock (Table 5).

## 4 Discussion

*C. psittaci* is an obligate intracellular Gram-negative pathogen commonly found in birds and poultry. Humans can become infected through direct inhalation or indirect exposure to infected bird secretions, aerosols of dry feces, or feather dust [4]. Pneumonia caused by *C. psittaci* has been reported to account for 1% of CAP in adults [3]. The exact incidence and prevalence of *C. psittaci* are difficult to determine due to the lack of routine testing and diagnostic methods [10]. With the development of detection technology and the popularity of mNGS, the incidence of *C. psittaci* is gradually increasing. The source of infection is not only limited to psittacidae birds but also includes poultry, etc. People become sick after contact with infected birds or birds [11]. All ten patients considered in this study have a history of recent exposure, among which four patients have a history of exposure to birds (parrots, pigeons, swallows), and six patients have a history of exposure to poultry (chickens, ducks). Therefore, for patients with a history of contact with birds or infected birds, it is necessary to be alert to the possibility of infection with atypical pathogens, especially *C. psittaci*.

At present, domestic and foreign literature studies have also reported cases of *C. psittacosis* pneumonia [12,13], and the clinical manifestations and severity of the disease vary. Therefore, we collected the data of ten patients with severe *C. psittaci* pneumonia in our hospital in the last 2 years and found that their clinical characteristics were different from those reported in the past. Previous studies have reported cases without ventilator support. The patients we included all started with high fever or diarrhea, accompanied by general symptoms such as cough, less phlegm, limb weakness, and dizziness. The disease progressed rapidly, and soon respiratory failure occurred, and the progression to severe pneumonia required ventilator-assisted ventilation treatment. This is similar to that reported by Chen et al. [6]. However, in general, the clinical symptoms of *C. psittaci* infection are not specific, and other laboratory and imaging evidence should be combined to support a definite diagnosis.

It has been reported in the literature [14,15] that patients with psittacosis have a normal WBC with a “left shift of the nucleus” and a significantly elevated CRP. In this study, only one patient had slightly higher WBC, and the rest had normal WBC. *N*% and PCT were increased in all patients, and CRP was significantly increased. This may be related to the greater pathogenicity of *C. psittaci* than other *Chlamydia* [16]. And because the included patients were all in severe condition, they were in the stage of systemic inflammatory response in the early stage of the disease, so inflammatory indicators such as CRP were significantly increased. Eight patients had abnormal ALT, AST, CK, and LDH, and the results were similar to those of Chen et al. [2]. Patients with severe infection are accompanied by endotoxemia, and a large number of inflammatory mediators are released into the blood, which leads to the imbalance of pro-inflammatory and anti-inflammatory bodies, resulting in incomplete metabolism of multiple organs in the body, and then abnormal liver enzymes, cardiac enzymes, and other indicators [17].

Image characteristics of parrot fever are not uniform. Studies have shown that unilateral lower lobe consolidation is more common on chest CT in patients with *C. psittacosis* pneumonia, which can develop into bilateral consolidation later [18]. Itoh et al. [19] found that the infection of *C*. *psittacosis* is dominated by ground-glass opacities, bilateral lung lesions are more common, bronchopneumonia and small nodules are rare, and a small number of patients have pleural effusion. Chest CT showed that some lesions had consolidation nodules and ground-glass degeneration. After the disease improved, the lesions on the chest CT could be completely absorbed without interstitial fibrosis [15]. In this study, most of the patients on the chest CT mainly showed patchy dense opacities involving multiple lobes on both sides. The majority of patients presented with small bilateral pleural effusions, which differs from previous studies. The reason may be that the patients included in this study were all critically ill patients, and most of the patients were transferred to our hospital for diagnosis and treatment. In general, there is no obvious specificity in imaging of *C*. *psittacosis* pneumonia, but the combination of clinical features and laboratory data can still accurately identify *C. psittacosis* infection.

In recent years, with the continuous development of mNGS technology, mNGS has been widely used in infectious diseases. Atypical pathogens in the lungs such as *Pneumocystis jirovecii*, *Legionella*, etc. can often be found [20,21]. Previous studies have not found the presence of *C. psittaci* contamination or background bacteria in respiratory specimens [22]. Therefore, once the sequence is detected, *C. psittaci* infection needs to be considered. In this study, the sequence of *C*. *psittacosis* was detected by mNGS in the bronchoalveolar lavage fluid of ten patients, and severe *C*. *psittacosis* pneumonia was diagnosed based on epidemiology and clinical manifestations of exposure history. The average time from admission to the detection of *C. psittaci* was 6.5 days, and the detection time was related to the severity of the patient’s condition, the effect of empirical treatment, and the willingness of family members. In addition, mNGS can also identify other bacterial and viral infections in critically ill patients. In addition to the detection of *C. psittaci*, the mNGS results in this study also detected pathogens such as *Corynebacterium gluconolytica*, *Klebsiella pneumoniae*, and *Aspergillus fumigatus*, which need to be combined with clinical manifestations and imaging examinations, and laboratory results were further confirmed to be the causative pathogen.

*C. psittaci* belongs to the *Chlamydia* family [23], and tetracyclines, macrolides, and quinolones that interfere with DNA and protein synthesis can be selected as antimicrobial therapy [24]. The first choice for the treatment of *C*. *psittacosis* is tetracycline antibiotics, mainly DOX. If there are contraindications, macrolides are the second choice. Fluoroquinolones also have a certain effect on psittacosis but much lower than the first two categories. Eight of the ten patients included in this study were treated with DOX after the diagnosis of *C. psittaci* pneumonia, and one patient was not treated with TGC according to CAP guidelines before the presentation of mNGS results. Fever and the re-examination of the infection indicators gradually returned to normal, so DOX was not switched. In this study, eight patients were diagnosed with *C. psittaci* infection and adjusted their anti-infection regimen in time, and they were eventually cured and discharged. One patient died of septic shock due to secondary bacteremia, and the other patient died of septic shock due to older age and respiratory failure that could not be corrected after tracheal intubation and ventilator-assisted ventilation. Eventually, death occurred before mNGS results were reported. According to clinical experience, pneumonia onset with high fever, especially patients with a history of contact with poultry or birds, should be highly suspected of infection with atypical pathogens such as *C. psittaci* when the treatment effect of broad-spectrum antibiotics is poor and should be treated as soon as possible with DOX. In general, targeted treatment of *C. psittaci* pneumonia has a good prognosis, while for critically ill patients with a prolonged disease course, bacterial and fungal infections may occur secondary to complications, and the prognosis is poor.

The limitation of this study is that it only included ten patients with severe *C*. *psittacosis* pneumonia, and the sample size is small enough to study all the characteristics of *C*. *psittacosis* pneumonia. This is a retrospective study, and no other detection methods such as PCR, serological testing, and sputum culture were used to confirm the diagnosis [25]. Our hospital is the central hospital of the Yuxi area, and most of the patients admitted are critically ill patients or those who have been referred from surrounding hospitals. Therefore, the results of the study may be more severe.

## 5 Conclusion

*C. psittaci* pneumonia is increasingly becoming common in clinical practice. The disease progresses rapidly, and it is easy to develop into severe pneumonia and even combined with multiple organ dysfunction. When the clinical manifestations of pneumonia patients are fever and dry cough, it is necessary to carefully ask whether there is a history of contact with birds and poultry. mNGS examination can shorten the diagnosis time, and tetracycline drugs such as DOX can be added as soon as possible. In the later stage, large-sample multi-center research is needed to guide clinical diagnosis and treatment.

## References

[1] Cao B, Huang Y, She DY, Cheng QJ, Fan H, Tian XL, et al. Diagnosis and treatment of community-acquired pneumonia in adults: 2016 clinical practice guidelines by the Chinese Thoracic Society, Chinese Medical Association. Clin Respir J. 2018;12(4):1320–60. 10.1111/crj.12674 PMC7162259 28756639

[2] Huang W, Hu S, Zhu Y, Liu S, Zhou X, Fang Y, et al. Metagenomic surveillance and comparative genomic analysis of Chlamydia psittaci in patients with pneumonia. Front Microbiol. 2023;14:1157888. 10.3389/fmicb.2023.1157888 PMC10265514 37323913

[3] Hogerwerf L, De Gier B, Baan B, Van Der Hoek W. Chlamydia psittaci (psittacosis) as a cause of community-acquired pneumonia: a systematic review and meta-analysis. Epidemiol Infect. 2017;145(15):3096–105. 10.1017/S0950268817002060 PMC9148753 28946931

[4] Hocking JS, Geisler WM, Kong FYS. Update on the epidemiology, screening, and management of Chlamydia trachomatis infection. Infect Dis Clin North Am. 2023;37(2):267–88. 10.1016/j.idc.2023.02.007 37005162

[5] Zhang H, Zhan D, Chen D, Huang W, Yu M, Li Q, et al. Next-generation sequencing diagnosis of severe pneumonia from fulminant psittacosis with multiple organ failure: a case report and literature review. Ann Transl Med. 2020;8(6):401. 10.21037/atm.2020.03.17 PMC7186658 32355845

[6] Chen X, Cao K, Wei Y, Qian Y, Liang J, Dong D, et al. Metagenomic next-generation sequencing in the diagnosis of severe pneumonias caused by Chlamydia psittaci. Infection. 2020;48(4):535–42. 10.1007/s15010-020-01429-0 PMC7223968 32314307

[7] Yang WS, Kang HD, Jung SK, Lee YJ, Oh SH, Kim YJ, et al. A mortality analysis of septic shock, vasoplegic shock and cryptic shock classified by the third international consensus definitions (Sepsis-3). Clin Respir J. 2020;14(9):857–63. 10.1111/crj.13218 32438528

[8] Mandell LA, Wunderink RG, Anzueto A, Bartlett JG, Campbell GD, Dean NC, et al. Infectious Diseases Society of America/American Thoracic Society consensus guidelines on the management of community-acquired pneumonia in adults. Clin Infect Dis. 2007;44(Suppl 2):S27–72. 10.1086/511159 PMC7107997 17278083

[9] Balsamo G, Maxted AM, Midla JW, Murphy JM, Wohrle R, Edling TM, et al. Compendium of measures to control Chlamydia psittaci infection among humans (Psittacosis) and pet birds (Avian Chlamydiosis), 2017. J Avian Med Surg. 2017;31(3):262–82. 10.1647/217-265 28891690

[10] Rybarczyk J, Versteele C, Lernout T, Vanrompay D. Human psittacosis: A review with emphasis on surveillance in Belgium. Acta Clin Belg. 2020;75(1):42–8. 10.1080/17843286.2019.1590889 30882289

[11] Shi Y, Chen J, Shi X, Hu J, Li H, Li X, et al. A case of Chlamydia psittaci caused severe pneumonia and meningitis diagnosed by metagenome next-generation sequencing and clinical analysis: a case report and literature review. BMC Infect Dis. 2021;21(1):621. 10.1186/s12879-021-06205-5 PMC8243071 34193063

[12] Deng F, Lin Q, Xu X, Li C, Xu J, Nie H. A case report of healthcare-associated psittacosis. J Infect Dev Ctries. 2023;17(4):571–7. 10.3855/jidc.17241 37159883

[13] Chau S, Tso EY, Leung WS, Fung KS. Three cases of atypical pneumonia caused by Chlamydophila psittaci. Hong Kong Med J. 2015;21(3):272–5. 10.12809/hkmj144321 26045070

[14] Spoorenberg SM, Bos WJ, van Hannen EJ, Dijkstra F, Heddema ER, van Velzen-Blad H, et al. Chlamydia psittaci: A relevant cause of community-acquired pneumonia in two Dutch hospitals. Neth J Med. 2016;74(2):75–81. 26951352

[15] Huang Y, Zheng W, Gan W, Zhang T. Chlamydia psittaci pneumonia: A clinical analysis of 12 patients. Ann Transl Med. 2023;11(3):144. 10.21037/atm-22-6624 PMC9951019 36846017

[16] Knittler MR, Sachse K. Chlamydia psittaci: Update on an underestimated zoonotic agent. Pathog Dis. 2015;73(1):1–15. 10.1093/femspd/ftu007 25853998

[17] Ziesmann MT, Marshall JC. Multiple organ dysfunction: The defining syndrome of sepsis. Surg Infect (Larchmt). 2018;19(2):184–90. 10.1089/sur.2017.298 29360419

[18] Heddema ER, van Hannen EJ, Duim B, de Jongh BM, Kaan JA, van Kessel R, et al. An outbreak of psittacosis due to Chlamydophila psittaci genotype A in a veterinary teaching hospital. J Med Microbiol. 2006;55(Pt 11):1571–5. 10.1099/jmm.0.46692-0 17030918

[19] Itoh I, Ishida T, Hashimoto T, Arita M, Osawa M, Tachibana H, et al. [Chest radiograph of atypical pneumonia: Comparison among Chlamydia pneumoniae. Pneumonia, ornithosis, and Mycoplasma pneumoniae pneumonia]. Kansenshogaku Zasshi. 2000;74(11):954–60. 10.11150/kansenshogakuzasshi1970.74.954 11140079

[20] Zhang Y, Ai JW, Cui P, Zhang WH, Wu HL, Ye MZ. A cluster of cases of pneumocystis pneumonia identified by shotgun metagenomics approach. J Infect. 2019;78(2):158–69. 10.1016/j.jinf.2018.08.013 30149030

[21] Huang Y, Ma Y, Miao Q, Pan J, Hu B, Gong Y, et al. Arthritis caused by Legionella micdadei and Staphylococcus aureus: metagenomic next-generation sequencing provides a rapid and accurate access to diagnosis and surveillance. Ann Transl Med. 2019;7(20):589. 10.21037/atm.2019.09.81 PMC6861802 31807570

[22] Miao Q, Ma Y, Wang Q, Pan J, Zhang Y, Jin W, et al. Microbiological diagnostic performance of metagenomic next-generation sequencing when applied to clinical practice. Clin Infect Dis. 2018;67(suppl_2):S231–40. 10.1093/cid/ciy693 30423048

[23] Sachse K, Bavoil PM, Kaltenboeck B, Stephens RS, Kuo CC, Rossello-Mora R, et al. Emendation of the family Chlamydiaceae: Proposal of a single genus, Chlamydia, to include all currently recognized species. Syst Appl Microbiol. 2015;38(2):99–103. 10.1016/j.syapm.2014.12.004 25618261

[24] Cantor A, Dana T, Griffin JC, Nelson HD, Weeks C, Winthrop KL, et al. Screening for Chlamydial and Gonococcal Infections: Updated Evidence Report and Systematic Review for the US Preventive Services Task Force. JAMA. 2021;326(10):957–66. 10.1001/jama.2021.10577 34519797

[25] Gu L, Liu W, Ru M, Lin J, Yu G, Ye J, et al. The application of metagenomic next-generation sequencing in diagnosing Chlamydia psittaci pneumonia: A report of five cases. BMC Pulm Med. 2020;20(1):65. 10.1186/s12890-020-1098-x PMC7077129 32178660

